# “Not everyone can do this”: childcare context and the practice of skill in emotional labor

**DOI:** 10.1186/s40723-022-00101-4

**Published:** 2022-08-08

**Authors:** Ragini Saira Malhotra

**Affiliations:** University of Southern Maine, Portland, USA

**Keywords:** Early Childhood Education and Care (ECEC), Childcare, Care work, Emotional labor, Gender, Skill

## Abstract

Scholarship remains divided about whether emotional labor is ‘skilled’. Interrogating gendered skill constructs that render emotions in work invisible, I examine two organizational contexts in the Early Childhood Education and Care (ECEC) sector: family and center-based care. I draw from 43 interviews, primarily with Latina and White women workers, reflecting feminized and racialized workplaces. I also draw from ethnographic and observational data. Challenging the particular devaluation of family-based care, findings reveal that the practice of skill in emotional labor is organizationally shaped across less and more institutionalized forms of ECEC. Examining worker critiques of professionalization norms and credential-based skill metrics, autonomy is also identified as a pre-requisite for embodied, tacit and discretionary skills in the emotional labor of ECEC.

## Introduction

### Background and context

Early Childhood Education and Care (ECEC) work is typically treated as “unskilled” in the United States and many parts of the world (Duffy et al., 2015; Fairchild & Mikuska, 2021). Yet, what constitutes “skill” is a source of debate, and associated skill constructs that devalue caring labor remain contested (Attewell, 1990; Bolton, 2004). Gendered presumptions about women as “naturally” good caregivers persist because of the association of motherhood and reproduction with care (Duffy et al., 2015; Fairchild & Mikuska, 2021; Stone, 1990). This naturalization of care means that despite being particularly time-intensive, ECEC is associated with the highest wage penalty relative to other care occupations, reinforcing market-based gender inequalities. In the United States, center-based care is considered more professionalized than family-based care; both program types are part of the country’s ECEC system (England et al., 2002; Folbre, 2001; Tuominen, 2003; Uttal, 2002). The latter is therefore uniquely devalued in the market; family-based care in the U.S. has been historically associated with lower skills and costs than center-based care because of its particular association with mothering and its location within the home (Armenia, 2006, 2009; England et al., 2002; Folbre, 2001; Tuominen, 1998, 2003; Uttal, 2002).^1^ These distinct contexts of care are therefore perceived differently and represent diverging levels of regulation and institutionalization.

The ECEC sector represents only one occupational space in a larger feminized care economy; its devaluation is inseparable from the gendered social construction of skill (England, 2005; England et al., 2002; Nixon, 2009; Tuominen, 2003). Presumed ‘natural’ characteristics of empathy and patience perpetuate misplaced perceptions about paid ECEC work as “unskilled”, manifesting in its’ inadequate recognition (Duffy et al., 2015). These notions also drive the invisibility of emotional labor, a central feature of caring and feminized professions (Fairchild & Mikuska, 2021; Gray 2010; Henderson, 2001; Mikuska & Fairchild, 2020; Hoschild, 1983). The skills and emotional labor in this work typically go unrecognized, thereby driving gender segregation and market-based inequalities (Fairchild & Mikuska, 2021; Hochschild, 1983; Mikuska & Fairchild, 2020).

With this as context, this paper contributes to ongoing scholarly debates about emotional labor, skills and the socio-cultural construction of professionalization across ECEC contexts (Bolton, 2005; Cohen, 2010; Ed. Langford, 2019; Fairchild & Mikuska, 2021; Mikuska & Fairchild, 2020; Morris, 2021; Osgood, 2010; Payne, 2009; Taggart, 2011; Vincent & Braun, 2013). Specifically, the paper demonstrates that in both family and center-based care settings, work *is* skilled. These skills are *learned*, not natural, and they are embedded in the performance of emotional labor and their practice is *organizationally shaped* across family and center-based care. Thus, building on existing scholarship, this paper demonstrates that the employment context of care matters and can shape differing requirements for emotional labor (Cohen, 2010; Lopez, 2010; Payne, 2009; Rodriquez, 2014; Vincent & Braun, 2013). Variations across these differently institutionalized forms of care—family and center-based—therefore lie not in the degree of skill, but in its *practice*.

These organizational variations^2^ complicate understandings of gendered skill in emotional labor, illustrating that family-based care is no less skilled than more institutionalized forms of ECEC work. This is an important contribution of the paper as family-based care has been particularly understudied and qualitative research on different ECEC contexts is even rarer (Armenia, 2006; McNeil, 2012; Nelson & Lewis, 2016; Tuominen, 2003). In several other ways, too, research at the intersection of these debates is timely from both a scholarly and policy perspective. Theoretically, this paper contributes to a re-emergent body of scholarship on care labor, emotional labor, and professionalization, within and beyond the United States. In particular, it extends this literature to focus uniquely on skills in emotional labor as they are organizationally shaped and professionally constructed across family and center-based ECEC contexts (Cohen, 2010; Lopez, 2010; Payne, 2009; Rodriquez, 2014; Vincent & Braun, 2013). In doing so, the paper examines critiques of credentialing requirements for ECEC workers in Massachusetts that equate higher skills with improved quality care and obscure skills in emotional labor (Bolton, 2005; Cohen, 2010; Ed. Langford, 2019; Fairchild & Mikuska, 2021; Mikuska & Fairchild, 2020; Morris, 2021; Osgood, 2010; Payne, 2009; Taggart, 2011; Tuominen, 2002; Uttal, 2002; Vincent & Braun, 2013).

Beyond its scholarly contributions, this paper’s findings can be leveraged to inform policy debates about the limitations of ECEC professionalization norms from the perspective of ECEC sector workers themselves. Findings shed light on workers’ own experiences and critiques, and emphasize the role played by distinct care work contexts in shaping requirements for emotional skills. The empirical analyses presented in this paper is therefore particularly relevant given the ongoing tension between professionalization standards across ECEC sectors and efforts to give recognition and valuation to the needs, labor, and skills of ECEC workers.

So, what, then, do these practices of skill in emotional labor look like? This paper finds that across both family and center-based care, workers possess more autonomy than scholarship suggests.^3^ They use judgment and discretion when performing what Arlie Hochschild (1983) first termed emotional labor. As teachers and providers juggle multiple tasks spontaneously, they manage emotions within themselves, in parents, and children. Importantly, however, their autonomy is not synonymous with skill. Instead, worker autonomy is a pre-requisite for the embodied, tacit and discretionary skills exhibited through emotional labor. These skills, often obscured, can be understood best through the feedback loop between task complexity, experiential skill acquisition and autonomy.

## Review of literature

### Care-work, skill, and professionalism

The feminization of care work is primarily rooted in the gendered construction of women as naturally better caregivers than men. Assumed to have ‘special abilities’, women have been principally responsible for unpaid care, historically devalued labor unrecognized as ‘real work’ (Cancian & Oliker, 2000; Duffy et al., 2015; Folbre, 2001; Mikuska & Fairchild, 2020; Nixon, 2009). Even when commodified, care is cheapened. Despite its economic contributions, the ‘care economy’ remains invisible in U.S. statistics, explaining its ‘debased’ nature (Armenia, 2006; Folbre, 2001). Some ECEC workers recognize their competence, identifying with ‘professionals’ and disassociating with motherhood and less ‘skilled’ care-workers like babysitters (MacDonald, 2010; Nelson, 1990; Nelson & Lewis, 2016; Tuominen, 2003; Uttal, 2002; Vincent & Braun, 2013). Yet gendered conceptions about work continue to shape and deepen gender inequalities across care and non-care occupations. These gendered dynamics, characteristic of feminized care occupations—and arguably service work more broadly—can also be reflected in workers’ gendered attitudes. A study of working-class men in the U.K., for example, demonstrates a reluctance on their part to engage in work involving emotional labor, patience, and the act of ‘put[ting] a smiley face on’ (Nixon, 2009).

Skill in workplaces has been defined in contradictory ways (Bolton, 2004; Grugulis et al., 2004; Hurrel et al., 2013; Payne, 2009). Conventional skill measures typically privilege expertise acquired through credentials or experience, under the assumption that education is the best path to skill development. Attention is therefore focused on credentials (Bolton, 2004; Brown et al., 2003). Classic operational skill measures—stressing work complexity and variety, as well as workers’ discretion, autonomy, control and power—ignore many dimensions of skill due to gendered assumptions (Bolton, 2004; Hurrel et al., 2013; Payne, 2009; Wheatley, 2017). This reflects deep biases contributing to perceptions of jobs typically associated with ‘feminine traits’ as ‘unskilled’ (Acker, 1989; Cockburn, 1983; Duffy et al., 2015; Grugulis & Vincent, 2009; Steinberg, 1990).

Although jobs performed by women and men are equally complex, employment evaluations ignore or assign negative values to emotional and interpersonal aspects of caring or ‘nurturant’ skills, conversely assigning positive values to ‘masculine-identified skills’. While ‘soft skills’—like self-management, interpersonal and service orientation skills—are increasingly recognized as critical in hiring and advancement, they are viewed as personality-based, not experiential (Bolton, 2004; Hurrel et al., 2013; Leidner, 1993; Payne, 2009). Critiques of conventional skill measures highlight the hyper-masculinized construction of skill; there is unpredictability even in routinized work requiring workers to use discretion and ‘know-how’ even in ‘unskilled’ workplaces (Juravich, 2009; Leidner, 1993).

Just as the idea of ‘skill’ is socially constructed, as Jayne Osgood (2010) shows us, so too is ‘emotional professionalism’. According to Osgood (2010), the socio-cultural construction of professionalism reflects neo-liberal ideologies that promote standardization and devalue emotional labor and capital rather than centering them as vital and credible. Others, too, have written about similar dynamics characterizing constructed professionalization norms in the ECEC sector (Bolton, 2005; Cohen, 2010; Ed. Langford, 2019; Fairchild & Mikuska, 2021; Mikuska & Fairchild, 2020; Morris, 2021; Payne, 2009; Taggart, 2011; Vincent & Braun, 2013). In the ECEC sector, credentialing requirements which have been associated with enhanced service quality, have been a central component of professionalization efforts. However, credentialing does not necessarily translate into higher quality (Armenia, 2006; Early et al., 2007; Folbre, 2012; Nelson & Abel, 2000; Uttal, 2002). High-quality care is associated more with flexibility, autonomy, nurturance, stimulating interactions, emotional care and trust (Cain, 2017; Duffy et al., 2015; Uttal, 2002). Conflating credentials with skills, is therefore problematic (Bolton, 2004; Payne, 2009). Bolton (2004) argues that by over-emphasizing certification we invisibilize ‘inarticulate’ forms of embodied knowledge, obscuring how skill acquisition is embedded in social relations.

### Complicating narratives on emotional labor and skill

Scholarship is divided on whether there is skill in emotional labor (Bolton, 2004; James, 1989; Korczynski, 2005; Payne, 2009). Emotional labor refers to how workers manage their emotions, consistent with organizationally mandated feeling rules (Hochschild, 1983). Hochschild (1983) presents three requirements for emotional labor: voice-to-voice/face-to-face client contact, production of an emotional state in others and employer control over workers’ feelings. Here, emotional labor is labor-power, and emotions managed at work are sold for a wage. Hochschild’s (1983) research examines the smiles of flight attendants and the anger of bill collectors, demonstrating how management appropriates workers’ emotions. To ensure customer satisfaction, employers require workers to conceal and generate emotions through surface acting (feigned display of ‘right’ feelings) and deep acting (generating ‘right’ feelings through self-deception). These processes can be gendered, as women, more than men, are expected to ‘surface act’ or feign caring feelings (Polletta et al., 2016; Vincent & Braun, 2013).^4^ Where emotional labor is required, de-skilling becomes inevitable as management’s standardization of emotions excessively constrains worker autonomy (Hochschild, 1983). Yet Hochschild (1983) herself notes the concept of emotional labor does not fully extend to all jobs; day-care is a case in point. Here, workers’ emotions, not directly supervised, are self-monitored based on client expectations and professional norms (Hochschild, 1983).

Despite this caveat, most scholarship has applied the concept expansively to a range of occupations including ECEC work (Wharton, 2009). Yet recent studies in care/non-care occupations complicate this lens (Bolton & Boyd, 2003; Lopez, 2010; Rodriquez, 2014; Wharton, 2009). Rodriquez (2014) demonstrates that nursing home workers sometimes generate emotions that contradict organizational demands. Stone (1990) and Stacey (2011) argue that care-workers autonomously ‘define care on their own terms. They also navigate autonomy in other care and non-care occupations (Duffy et al., 2015; Lively, 2002; Sallaz, 2002). Specifically, Paules (1991) and Pierce (1996) explore how waitresses and litigators perform emotional labor, subverting client demands and rejecting cultural norms about gamesmanship, respectively.

Different organizational contexts of care shape the identity strategies workers use to protect their self-worth in the workplace (Nelson & Lewis, 2016). The way in which care organizations manage emotions, also varies across context in complex ways (Lopez, 2006). Lopez (2006) argues that care organizations can be positioned along a continuum, with those requiring emotional labor situated at the coercive end, and those requiring organized emotional care at the other. In three different settings Lopez (2006) finds evidence for: emotional labor; the co-existence of emotional care *and* emotional labor; and organized emotional care characterized by varying levels of autonomy and authenticity in worker–client relationships. *Organized emotional care* reflects the highest levels of autonomy and authenticity, including organizational support for ‘emotional honesty’ (Lopez, 2006). *Emotional care and emotional labor* balance organizational rules with authenticity. Breslin and Wood (2015) also find that informal rules shape care work, often in competition with more ‘formal’ rules.

Organizational variations in emotional labor exist even in non-care occupations. Cohen (2010) finds self-employed hairstylists are more likely to engage in ‘deep’ (not ‘surface’) acting than wage-based stylists. While neither Lopez (2006) nor Cohen (2010) focuses primarily on ‘skill’, others highlight often invisible experientially learned skills in emotional labor (Cain, 2017; Paules, 1996; Pierce, 1996; Rodriquez, 2014; Stacey, 2011; Vincent & Braun, 2013).

Scholars have also argued emotional labor fails to satisfy two key elements of skill—discretion/task complexity and control over the labor process (Payne, 2009). Others argue that emotional labor has been ‘deskilled’ (Ikeler, 2016). Yet, defending the skill in ‘organizational emotionality’ Bolton (2004) identifies workers as ‘multi-skilled emotion managers’. Claiming workers own the means of production and capacity to perform emotion (see Brook, 2009 for a critique), she argues Hochschild (1983) mistakenly conflates emotion work based on professional norms with commercialized emotional labor.

### Emotional labor and skill in ECEC

McNeil’s (2008) study of family and center-based care and Colley’s (2006) study of day-care trainees, demonstrate how workers rely upon an experientially based ‘unwritten curriculum’ of emotional bonding and a ‘hidden curriculum’ pertaining to the management of emotions through detachment. Studies from outside the United States, too, explore the role of emotions in ECEC work settings (Bolton, 2005; Cohen, 2010; Ed. Langford, 2019; Fairchild & Mikuska, 2021; Mikuska & Fairchild, 2020; Morris, 2021; Osgood, 2010; Payne, 2009; Taggart, 2011; Vincent & Braun 2013). Even studies on ECEC work not focused on emotional labor, reveal how workers navigate emotional and business aspects of work (Armenia, 2006; Tuominen, 2003; Uttal, 2002). As Morris (2021) notes, in the context of the ECEC sector, there is a requirement to navigate a dynamic tension between an embodied care ethic and the pragmatism of self-regulation. Family-based providers, for instance, traverse a ‘marketized private life’ (Hochschild, 1983) through boundary work^5^ and strategies of detached attachment (Nelson, 1990; Rodriquez, 2016). They might also develop genuine attachment bonds, invoking ‘family’ ideals, complicating the exchange of labor for wages (Duffy et al., 2015; Nelson, 1990; Tuominen, 2003; Uttal & Tuominen, 1999).

Through relational work (Zelizer, 2005) ECEC workers negotiate intimate and economic relations by establishing boundaries and minimizing role confusion. They respond to unpredictable, often unreasonable behaviors from parents *and* children (Folbre, 2001; Joffe, 1997; Tronto, 1993; Uttal, 2002; Zelizer, 2005). Workers challenge the strict ‘love or money’ dichotomy in ECEC work (Folbre & Nelson, 2000; Himmelweit, 1999; Nelson, 1999). Assumptions that this work must be motivated primarily out of ‘love’ are both gendered and racialized. It is assumed that women of color (specifically Latinas) possess less ‘skill’ because they are more likely than Whites to have ‘naturally’ caring dispositions (Duffy et al., 2015; Tuominen, 2003, 2008; Uttal, 2002). This article demonstrates that while the *practice* of skill in emotional labor varies across ECEC settings, the *degree* of skill does not differ across these contexts. This practice of skill is explored across a racially/ethnically diverse sample of caregivers in both care contexts, highlighting why recognizing skill in emotional labor is so crucial and how raced and gendered assumptions do not hold true.

## Methods

This study draws from 43 semi-structured interviews with family and center-based care-workers in Massachusetts, in combination with observational and ethnographic data. All but two interviews were conducted in workplaces, which allowed for concurrent labor process observations. I also conducted a six-week ethnographic study at a University campus ECEC center where I interviewed teachers. Although I often refer in this paper to family-based care providers and center-based teachers as workers, I also use language that reflects their self-identification as ‘providers’ and ‘teachers’. The latter as an identity is consistent with other scholarship on early childhood work (Nelson & Lewis, 2016; Tuominen, 2003).

Regulatory ECEC regimes vary across the United States, with higher levels of regulation being associated with greater quality of care. This study was conducted in Massachusetts, which has one of the most highly regulated (and therefore, costly) ECEC industries in the United States.^6^ Teacher–child ratios in ECEC centers, for example are 1:3 in Massachusetts, which is more favorable than most other states. There are two primary types of licensed child care provisioning in the state: (1) center-based and (2) family-based care. Centers serve children full-time or part-time in groups or classrooms. Children are typically grouped according to age with infants/toddlers in separate classrooms from nursery/pre-school aged children, for instance. Differently from centers, family-based care is delivered in provider’s homes. Children in family-based settings may range in age from infants to school-going aged children. Family-based settings may serve between a maximum of six or ten children (with an assistant permitted).^7^

With this as context, it is notable that the study coincided with the implementation of a state-wide policy designed to further increase quality across center-based and family-based settings through staff credentialing.^8^ The policy implicitly equated credentialing with higher skills and quality care, as well as increased professionalization. While not part of my initial design, this provision became central context for my research. It soon became clear that center-based teachers and family-based providers feared losing their jobs as they struggled to comply with policy stipulations. They shared their frustrations that State policy equated credentialing with higher skills. Their critiques reflected those made about the constructed nature of professionalism norms in varied ECEC contexts (Bolton, 2005; Cohen, 2010; Ed. Langford, 2019; Fairchild & Mikuska, 2021; Mikuska & Fairchild, 2020; Morris, 2021; Osgood, 2010; Payne, 2009; Taggart, 2011; Vincent & Braun, 2013).

When sampling cases, I maximized potential organizational variation to reflect diversity in family and center-based organizations, urban–rural contexts, and the race and ethnicity of workers. I used combined sampling strategies—respondent-driven, snowball and theoretical—to diffuse limitations associated with selecting similar cases (Wilson and Chaddha 2009). Workplaces were selected based on conversations with union representatives; persistent calls and written requests based on the ECEC state listings; and respondent-driven and snowball sampling.

Guided by the principle of saturation (Small, 2009), I conducted in-depth, semi-structured interviews with twenty center-based teachers, sixteen family-based providers, five center Directors, one administrative assistant, and the primary representative for the state Department for Early Education and Care (see Appendix, Table 1). Lasting 60–75 min on average, some were 2 h. I also conducted informational interviews with a Director and campaign coordinator for the state-wide Early Childhood Educators Union. I conducted four follow-up interviews, ranging from 30 to 75 min, to seek clarifications about points raised during initial meetings. Almost all interviews were in person and recorded^9^; two were in Spanish, not English.

I consider only licensed center and family-based care providers. Family-based interviewees were a mix of independent and agency-affiliated,^10^ and center-based interviewees represented eight different medium sized non-corporate centers, with similar degrees of institutionalization, regulation and capacity. To maximize my organizational comparison, each center (with one exception) is represented by two to three teachers. I also maximized potential variation by considering family and center-based care settings in the following areas of Western Massachusetts: Amherst, Northampton, Southampton, Belchertown, Greenfield, Holyoke, and Springfield. This geographic variation is closely associated with racial-ethnic variations. Consistent with existing studies that indicate, nationally, Latin X women are overrepresented in family-based care, I find each institutional context is racialized differently (Folbre, 2012; Lopez, 2010; Tuominen, 2003).^11^

Of my 43 respondents, 27 were White, fifteen self-identified as Latina/Hispanic, and specifically Puerto Rican immigrant, and one self-identified as Black (see Appendix, Table 2). Of the White interviewees, 15 were center-based teachers, six were family-based care providers, four were center-based Directors, one an administrative assistant, and one a State representative. Of the Latina interviewees, four were center-based teachers, and ten were family-based care providers, and one was a Director. The Black participant was a center teacher. Educational qualifications varied across and within context, as well as by race/ethnicity. All but one interviewee were women, a reflection of the gendered nature of the field.

Questions were modified for each setting, covering aspects of the labor process, a typical workday, interactions with parents/children, quality care and emotions. With Directors and the state representative, questions focused on policy logic and perceptions of ECEC work. All but two interviews were conducted at centers or family-based sites. My observations had greater breadth in family-based settings given the structure of work in this organizational context made routine nap times (standard in centers) difficult. Triangulating my interview, observational and ethnographic data, deepened my understanding of the labor process.

I received approval for this research from the Institutional Research Board (IRB). Data collection and analysis occurred concurrently. All but four^12^ interviews were transcribed and coded using NVivo. While I initially used deductive codes, as I advanced in data collection and analysis, I revised analytical categories based on inductively derived codes (Emerson 1995; Luker 2008; Marshall and Rossman 2011). My analytic themes on the labor process comprised separate codes for skill with parents and children, respectively, as well as a code for broader narratives on skill. The first two categories comprised sub-codes related to communication (including language and tone), patience, emotion management (including behavior), teaching and care, boundary work/attachment, and professionalism, amongst others. The broader skills code comprised sub-codes primarily spanning autonomy, experience, motherhood (including naturalization), credentialing, training, and valuation. Other important codes pertained to quality (related and unrelated to skill and the QRIS), institutional context (i.e., intra-context variation, institutional support), and non-skill characteristics.

## Findings

*‘The kids need my best self…you have to model like the appropriate reaction to any emotion that you’re feeling...like instead of getting angry…it’s very draining…Children have a range of emotion throughout the day that we have to cope with and manage and help them with…It’s a big part of what we do.’* ~ *Liz [White, center, 30s]*

This section contains analyses of the data collected for this paper. It is organized in terms of the following themes. First, a discussion of ECEC skills and what they look like. Second, what it means to learn skills experientially or on the job. This section includes workers’ critiques of professionalization standards and credentialing. The subsequent section maps out how practices of skill are organizationally shaped across family and center-based settings. Finally, given the differential requirements for skills used with children and parents, the last two sections address these areas separately. Through these sub-sections, skills are discussed in relation to emotional labor.

### ECEC skills

This section outlines the skills that teachers and providers identified as essential to their work. Interpersonal and emotional skills were given particular primacy, consistent with scholarship on the importance of emotions in ECEC work (Colley 2006; Fairchild & Mikuska, 2021; Vincent & Braun, 2013; Mikuska & Fairchild, 2020; Morris, 2018; Osgood, 2010; Elfer 2012, 2015; Ed. Langford, 2019).

As Martha, [White, center, 40 s] noted, ECEC work is ‘a job where you have to have lots of emotions.’ It requires embodied and experiential skills which are devalued societally and by the state. Caregivers emphasized the importance of experience in skill-building. They did not feel credentials were most important for the shaping of their most critical skills: managing emotions within themselves, children and parents (Colley 2006; Vincent & Braun, 2013; Elfer 2012, 2015). Teachers and providers emphasized the skills in the emotional labor of ECEC work, arguing that these are not acquired through credentialing and are undervalued by the state.

Managing emotions demands a range of skills. Patience is constructed as a skill; it is also a pre-requisite for effectively leveraging communication skills to manage children’s and parents’ emotions. In fact, Liz [White, center, 30s] stated her biggest challenge was to ‘cultivate patience’ while communicating with parents. Similarly, Alma [Latina-immigrant, family, 40s] noted ‘it’s harder working with the parents than the kids’—a sentiment echoed across settings. Ximena [Latina-immigrant, family, 40s], for example, felt you ‘need to be patient with parents more than with children’, and Rachel [White, center, 30s] noted that, ‘…the kids are the easy part’. She explained how she leveraged patience and judgment, conveying concerns to parents about their children’s behavior: ‘You want the parent right on board with you but they can’t always come along at the same rate. You have to be somewhat patient with what’s going on with them personally’. Thus, teachers and providers across settings, skillfully respond to parental concerns (Uttal, 2002) while managing their own emotions. Sometimes, says Jane [White, center, 40 s], you also have to be ‘a good politician with the parents’ especially when discussing sensitive issues like special needs. Lynne [White, center, 40 s] noted this requires patience, but also diplomatic management of ‘crying’ parents, often in denial about their child’s reality.

### Learning skills on the job

Teachers and providers rejected gendered understandings of their work as ‘natural’ and therefore ‘unskilled’. Many highlighted experientially acquired skills, challenging the presumed natural quality of paid work in the ECEC sector. Marisol [Latin-Immigrant, Centre, 40s] noted: ‘people *assume* because you're a woman you have the skills to be a teacher…you do *not…*the *first* time a child hit me with play-dough…I *cried*. I went home crying’. Such skills, required to manage emotions deemed inappropriate for the workplace, were identified as important across settings.

While some teachers and providers indicated you need ‘a certain amount’ of patience from the outset, almost all emphasized patience grows with experience. Carrie [White, Centre, 30s] points to these complexities in her work with children:

‘I did have a lot of it [patience] coming in but I’ve had to learn how to be *more* patient…teaching in a classroom…it’s changed my personality but it’s changed my skillset in terms of how I work with other people, how I communicate…using *words* to express feelings. Using *manner*…it’s a practice, it’s a practiced…*thing* that we do here every day.’

Patience is critical for the management of teachers’ own emotions. Far from being ‘innate’, this skill is acquired by ‘doing’, not from ‘a textbook’ [Sara, White, 20s]. Lindsay [White, center, 20s] noted: ‘In school you learn how to educate…[children] but you don’t learn how to…manage their *emotional*…kids are happy and the next thing you know two kids are *fighting*…you don’t learn that at school.’ This perspective, shared by several other teachers and providers, underscores how workers balance multiple techniques to manage emotions in others. They use discretion, making on-the-spot autonomous decisions in unpredictable situations.

Emphasizing experiential skills, workers begrudged having to ‘prove’ their competency through mandated professionalization that did not appear to guarantee increased remuneration. Worker critiques of the naturalization of care were inseparable from critiques of the state’s professionalization efforts. Though designed to raise the value of ECEC work as a profession almost all center-based teachers and providers viewed investments in education as a financial setback, fearing job loss when these were not possible.

Even when education was deemed valuable there was a tension between teachers’/providers’ conceptions of skill and the state narrative. Even those with formal qualifications stressed experience mattered more than credentials, or that the latter could not substitute for the former (Rodriquez, 2014). These perspectives were reflected in the data irrespective of educational status and are consistent with scholarship addressing the limitations of professionalization standards with regard to credentialing and quality (Armenia, 2006; Bolton, 2005; Cohen, 2010; Early et al., 2007; Ed. Langford, 2019; Fairchild & Mikuska, 2021; Folbre, 2012; Mikuska & Fairchild, 2020; Morris, 2021; Nelson and Abel 2000; Osgood, 2010; Payne, 2009; Taggart, 2011; Tuominen, 2003; Uttal, 2002; Vincent & Braun, 2013).

For example, Sara [White, center, 20 s], a college graduate, did not feel her degree should be valued over ‘hands-on-knowledge’, noting her colleagues resented having to ‘prove themselves’ to the state. Veteran teacher Ivonne [White, center, 50s] said, ‘I realize all I’m doing is *proving*…I’m proving what I’ve been doing all along’. Noting her Masters had not prepared her for the challenges of family-based care, Kimberly [White, family, 30s] indicated this work was ‘much more difficult than teaching’ in public schools. Similarly, Rachel [White, center, 30s] said: ‘I studied early Ed in school. I can't tell you *one* thing that I learned that would be relevant to this, except for *one* day…we learnt how to make special snowflakes’.

Similarly, Marisol [Latina-immigrant, center director, 40s], with a Master’s, remarked, it might be ‘…quicker to get the skills but just because you have a Bachelor’s degree doesn’t mean you can actually have those skills’. She stressed, ‘…the main skills develop through working’ and experience…[that’s] what *really* gives you the *ability’.* Thus, credentials cannot be equated with skill. While they might make skill acquisition faster, translating knowledge into requisite skills is not automatic. Carrie [White, center, 30s] echoed this:

‘…being in their [children’s] world…having that subset of skills, creativity mindfulness, thoughtfulness, umm thinking outside of the box, those are things you can't…I don't know if sitting through the classroom and you know going through the paperwork and doing the test would *make* me a better teacher.’

Rachel, who having obtained communication skills experientially, lead experiential-based trainings, made a similar point. Her autonomy in this process reflected the centers’ recognition of teachers’ expertise.

Yet, in most instances center-based teachers and providers did not feel their expertise or skills were valued (Gass, 2004; Uttal, 2002). Carrie said: ‘I do believe that the job we have is one of the most important jobs out there, um, I do *absolutely feel* like it is *not* valued enough by the government [and] just the general population.’ She noted that because people end up ‘devaluing’ ECEC work, center-based teachers feel they are ‘merely *babysitters’*. Reaffirming other scholarship (Fairchild & Mikuska, 2021; Nelson & Lewis, 2016; Tuominen, 2003) center-based teachers and family-based providers commonly dis-identified with ‘babysitters’. Gabby [White, center, 20s] noted, ‘Some of my friends are like, oh you’re babysitting, and I’m like, no…*I’m not babysitting…*I *teach* them’. Like Gaby, many workers across both ECEC settings identified their work as more legitimate, dis-identifying with babysitting.

### Organizationally shaped practices of skill

Contrary to conceptions about family-based care being less ‘skilled’ than center-based care which is considered to be more institutionalized (Folbre, 2001; Uttal, 2002), it is not skill degree that varies across organizational context, but its *practice.* Although family-based providers are self-employed, parents influence (but do not control) the practice of emotional labor—like employers, they extract workers’ emotional labor for a wage. While across settings workers have face-to-face client interactions, producing emotions in others by regulating their own feelings, neither family nor center-based care is characterized by rigid employer/client control. Autonomy not only allows for, but necessitates skill in the emotional labor of ECEC work, making it anything but ‘unskilled’. Irrespective of ethnicity/race, family-based care providers underscored nurturing aspects of care with accounts about skill in emotional labor. Consistent with Tuominen (2003), they adopted a ‘family style’ [Tina, White, family, 30 s] or ran their day-cares ‘like a family’ [Sofia, Latina-immigrant, family, 20s], but still viewed their work as skilled.

Center-based teachers (across race/ethnicity) emphasized a desire to care for children. They talked about making children’s ‘lives better’ [Tanya, White, center, 20s], wanting to ensure they were ‘comfortable’ [Rachel], rather than invoking family ideals. These accounts, more similar within settings than across ethnic/race-based groups, reveal organizational variations in care processes (Lopez, 2006; Stacey, 2011; Tuominen, 2003) and the practice of skill.

Neither setting represents coercive emotional labor or organized emotional care^13^ (Lopez, 2006). Instead, each reflects *emotional labor and care* to varying degrees. Hegemonic feeling rules are not dictated by management. Management does not enable the development of amicable relationships between service providers and recipients; a condition required of *organized emotional care* (Lopez, 2006). Although family-based care providers come closer to embracing elements of *organized care* (bonding during sleepovers) they do not engage in fully unfettered relationships with children or parents. It appears, therefore, that family and center-based care fall most accurately within the realm of *emotional labor and care* (Lopez, 2006).

Emotional labor is typically viewed as unskilled because workers who perform this work are perceived to lack autonomy over the labor process (Payne, 2009). While this article reveals the importance of autonomy, it does not equate it with skill, arguing instead, that autonomy is a pre-requisite for the performance of emotional labor, necessitating much of the complexity in this work.

This manifested differently across settings with some overlaps. Both center-based teachers and providers had ‘a lot of autonomy’ [Tara, White, center, 30s] and considerable ‘freedom’ in their work [Belinda, Latina-immigrant center, 30s; Sofia, Latina-immigrant, family, 20s]. However, in ECEC centers, worker autonomy was more clearly mediated by organizational requirements and norms about professional and appropriate behavior. While some teachers shaped norms about ‘behavior management’ and ‘how to talk to children’ [Rachel, White, center, 30 s], *all* family-based care providers would self-direct and define organizational norms autonomously (Stacey, 2011). These norms guided the practice of skill in emotional labor, which was shaped differently across organizational context.

Autonomy is often conflated with a lack of standards, ‘professionalism’ and even skill in family-based care because of its location in the home (Folbre, 2001; Tuominen, 2003). This article’s findings challenge this assumption. While center-based teachers were more likely to invoke ideas about professionalism than family-based providers, they noted workplace communication norms/feeling rules were not imposed by management (Hochschild, 1983). Susanne [White, center, 30] noted center-based teachers were ‘asked’ to observe communication norms—not *told* to do so. She emphasized there is no ‘…*list*, but there’s a definite…be aware of how you’re talking to kids…do it in a *positive* way…there's no, “say this and not *this”*…you hear it and you’re like, oh that works’. Many other teachers said communication norms were ‘informal’ and ‘unspoken’ allowing for worker agency (Lopez, 2006). Family-based providers also organized their workplaces using self-directed norms about appropriate communication. Tina [White, family, 30 s] said: ‘If I was sad or something I wouldn’t let the kids see it…It’s not for them’, indicating communication with children requires internal suppression and management of emotions (surface acting).

As sole representatives of organizations, family-based providers emphasized the burden of autonomy, noting ‘more independence’ means ‘more work’ [Patricia, Latina-immigrant, family, 40 s]. A Director of an ECEC center, and former family-based provider, remarked: ‘You *have* to almost have more skills’ [Ivonne, White, center, 50 s] when dealing with an age-diverse self-directed environment. Autonomy therefore enabled—or required—the display of tacit, embodied and discretionary skills. Most providers felt an absence of organizational support (or ‘accountable’ directors) made work more challenging. Rosa [Latina-immigrant, family, 40 s] noted:

‘I think the skills are the same. I think what changes is the responsibility. [In ECEC centers]… the responsibility is shared between the principal and other teachers. Here I am the only one. If the child gets hurt it is my responsibility. If the child comes home sunburned, it is my responsibility. I have to have the ability to serve all children equally. Give food to them, to teach them. I have to have those skills.’

Having full responsibility for “juggling” diverse tasks concomitantly distinguished the practice of skill in emotional labor from centers (Bolton, 2001).

Indeed, some family-based providers believed it was consequently ‘easier’ working in centers. [Rosa, Latina-immigrant, family, 40 s]. Sofia [Latina-immigrant, family, 20 s] emphasized: ‘When you're working at home, you’ve got to do everything *yourself*. We're doing the diapering, we’re doing the cooking, we're doing the napping, we're doing the nursing, we’re doing *everything’*. Alma [Latina-immigrant, family, 40 s] reaffirmed this:

‘I *think* we are jack of all trades. We do a lot of things…we're nurses, we educate…we’re teachers, we're *mom,* we’re psychiatrist…a little bit of everything because if a child gets hurt, who's there to kiss their boo boo? We are. To put them to sleep, we are. To feed them, we are. We’re cooks…there’s only one person doing *everything…’*

Many echoed how the practice of embodied skill in emotional labor was shaped by workplace structure. Teachers juggling multiple roles received colleagues’ support^14^ and did not have to manage a crying child while cooking lunch, as Carrie’s [White, center, 30 s] experience revealed:

‘I think we have to wear multiple hats…when you talk to a child about…misbehaviour or discipline that is an entirely different set of tools that I have to use because that’s not time for me to be silly…that’s time to use a certain voice, to use different negotiation tactics and skills to figure out solutions, so even within the context of being a teacher, there are lots of different jobs within that…it takes skill to know which hat to wear.’

This highlights differences in roles performed by family-based providers and center-based teachers. The ‘multiple hats’ worn by teachers—while skilfully selected/discarded situationally—pertained more narrowly to classroom management. Workplace structure shaped the practice of skill in emotional labor distinctly with parents too. Across ECEC settings, discretion and task complexity characterized skill in emotional labor.

### The practice of skill in emotional labor with children

Across ECEC settings, workplace-specific strategies were used in performing emotional labor with children. Sara [White, center, 20 s] noted her trainee experience taught her while working with children you must: ‘Maintain your own balance…when I got really crazy that showed…my verbal communications and my non-verbal communications were off…weren't appropriate…I started unfortunately like yelling at the kids’. This underscores the demands of surface acting (suppressing anger and frustration), guided by implicit organizationally institutionalized norms. Rachel [White, center, 30 s] elaborated: ‘…you try to keep your…cool…inside your head you’re like “oh my god, like argghhh” if they do it one more time…’. Each morning, Rachel said, she took a deep breath saying, ‘OK, I can do this’. She recalled a conversation with an observer of her classroom:

‘This is your break? And I was like, I just I just need some quiet time. She's like, what's going on? I was like it’s just a *crazy* morning, she was like, you were so *calm* and I was like, if you knew what was going inside my head (laughs)…’

These accounts suggest Susanne and Rachel engage in surface acting (Hochschild, 1983); modified facial expressions reflecting workplace ‘feeling rules’ shaped by implicit, non-imposed norms about professionalism. Teachers ‘juggle’ multiple roles, managing their emotions and others’, consistent with literature on skill in service work (Bolton, 2001).

Lindsay [White, center, 20s] reported having to ‘put on that happy face’ while being ‘strict’ and ‘serious’, while Carrie [White, center, 30s] noted that, ‘…balancing that respect’ and affection is ‘absolutely…a skill’. Similarly, Gabriella [Latina-immigrant, center, 20s] remarked: ‘You gotta show them that you're …the one in control not them…[you] also wanna balance that with *sweetness*… I’m always smiling to them…*always…’.* Given children’s unpredictable behavior, tacit and discretionary skills are required to balance authority with a ‘caring’ disposition.

This is also true in family-based care. Alma [Latina-identified, family, 40s] noted when children were upset she used to ‘cry with them’, while today she can ‘control’ children’s emotions, running her day-care on her own terms. The age-diverse nature of family-based care also shaped the practice of skill distinctly from centers. For instance, Amy [White, family, 30s] reflected on her experience in public schools: ‘I feel like people don’t understand how hard this job is, it’s much more difficult than teaching ‘cos the age groups are all different…you have the babies that just cry. It’s exhausting’. With a toddler in her lap and an infant in her arms, during our interview, I watched Amy anticipate and ‘juggle’ unpredictable behaviors of children of varied ages. She stressed that ‘being able to address their emotional needs’ simultaneously and without assistance, required ‘*so* much managing’ and ‘so many skills’. Yet, she obscured her frustration by surface acting, maintaining calm in tone and behavior.

Teachers and providers exercised discretion about when to ‘perform’ or genuinely display smiles. During classroom observations, I watched Susanne [White, center, 30s] react to the stress of managing children during a challenging naptime. Retreating briefly to her office space, Susanne’s smiles vanished. She took a pill, speaking about how challenging her group had been; yet she kept cool amongst children. Family-based providers balanced emotions differently; since workplace structure offered ‘no relief’ or ‘anyone to talk to’ [Amy, White, family, 30s], isolation would sometimes lead to post-work breakdowns [see Cohen, 2010; Vincent & Braun, 2013)].

Teachers used institutionalized boundary work as a strategy; family-based providers performed self-directed boundary work. Both used bodily communication (exaggerated head shakes, disapproving looks) to distance emotionally from children. For example, Liz [White, center, 30 s] recalled using reflexive discretion to discourage tantrums:

‘I think about the *loooonnnng* battle I had with someone who was having a meltdown, knowing when you have to walk away…wondering when are they going to be ready to, to come talk about it, that discipline always feels like...the most exhausting part of the day.’

Setting boundaries therefore requires managing emotions internally and in children simultaneously:

‘A common strategy is to ignore the behavior to some extent...I'm not emotionally involved...I am and I do get like frustrated and upset sometimes...people tell me I don't look upset but I am (laughs)…it’s a skill…to be able to do that and not let the kids always *see* that you're feeling that frustrated.’ [Liz, White, center, 30s]

This form of surface acting, guided by norms about professional workplace culture, required teachers to use ‘detached attachment’ strategies (Nelson, 1990; Zelizer, 2005).

Teachers said they must ‘kind of be happy’ for children when they move on, as telling them they will be missed is ‘not going to help’ [Dasha, White, center, 40 s]. While providers did not experience these transitions as often, they also managed feelings of ‘withdrawal’ [Kendra, White, family, 50 s]. While some reported blurring boundaries (kissing, attending birthdays) like in centers—in Rachel’s [White, center, 30 s] words—most established ‘some kinda distance’. Across centers and race/ethnicity, as expressed by Rachel, ‘…it’s hard to distance yourself…you love them [kids]…[but] you’re *not* their parents, you can’t…fix everything in their lives’. Therefore, irrespective of race/ethnicity, only some teachers felt like ‘second mothers’. This was also true of providers who sometimes identified as ‘grandmothers’ [Rosanne, family, 40 s] or an ‘extension’ of parents [Tina, White, family, 30 s]. As Morris (2021) notes, ECEC work requires navigating the tension between an embodied care ethic and the pragmatism of self-regulation. Doing so, requires what Jools Page (2018) calls “professional love”. This act of balancing ‘love’ and boundary work to avoid role confusion (Nelson, 1999; Zelizer, 2005) requires considerable skill.

These requirements for skill, elaborated upon below, are in tension with the way in which ideas about professionalism have been constructed in ECEC sectors. As Taggart (2011) notes, for instance, in the U.K. discourses of professionalism exclude an ‘ethical vocabulary of care’ despite relying on workers’ gendered dispositions towards emotional labor. On the one hand, the skillful display and management of emotions are part of the job, and on the other hand, standards of professionalism don’t value these emotions. This dynamic reinforces the critiques of credentialing requirements leveraged by ECEC workers in this paper, consistent with scholarship on the constructed nature of professionalism norms in the industry (Bolton, 2005; Cohen, 2010; Ed. Langford, 2019; Fairchild & Mikuska, 2021; Mikuska & Fairchild, 2020; Morris, 2021; Osgood, 2010; Payne, 2009; Taggart, 2011; Vincent & Braun, 2013). Navigating this tension can be disadvantageous for workers, placing requirements on them to perform an undervalued form of caring labor. In this context, the following section examines how practices of skill in emotional labor are organizationally shaped in family and center-based care settings when it comes to interactions with parents in particular.

### The practice of skill in emotional labor with parents

With parents, skill in emotional labor is performed uniquely across settings; its practice is shaped by parents’ distinct roles. Relational, boundary work (Zelizer, 2005) is required to manage unrealistic parental expectations. This perhaps reflects assumptions that care is motivated out of love, not money (Folbre & Nelson, 2000; Tuominen, 2003; Uttal, 2002), particularly for family providers and/or women of color. Yet, Alma [Latina-immigrant, family, 40 s] complicated this dichotomy by asserting that, ‘…I love the kids, you know, but I need the money’.

With parents, presenting ones ‘best self’ is key said Liz [White, center, 30 s]: ‘You can’t just say, “oh yeah I’m having a *crap* day” to a mom. She doesn’t want to hear that. You’re taking care of her precious *thing* all day long. She doesn’t want to know you had a bad day’. Strategies of institutionalized boundary work are therefore required to maintain authority, said Susanne:

‘You try to be professional and friendly...occasionally you have a parent act, inappropriately....had a parent like really yell at me…it was really not ok......that's like much more challenging to me than a child having a tantrum……you have to maintain professionalism...I did tell them it’s not ok to talk to me like that….sort of setting a boundary... I'm not going to yell at them…you have to maintain professionalism.’

Concealing negative emotions by performing neutral ones, Susanne simultaneously advocated for herself while observing norms of professionalism. She equalized unequal relations where, as a worker, she enjoyed less courtesy than parents (Hochschild, 1983; Wharton, 1990). Others, like Gabriella [Latina-identified, center, 20 s], experienced similar challenges:

‘I had a parent that she would talk very dry to you..I would just you know keep straight…smile….I'm trying to help you and I’m not your enemy, I’m your friend...you can't be *too* friendly neither….you have to keep it *professional…’*

When boundary-setting with ‘rude’, disrespectful parents, most teachers sought organizational support: ‘…we just go with the flow ‘cause we can’t argue with them…we talk with them and if they’re wrong…we go to the supervisor’ [Ana, Latina-immigrant, center, 40 s].

Family-based providers did not enjoy support for boundary work. For instance, Amy [White, family, 30 s] struggled to negotiate paid overtime with a client:

‘[She] is a *teacher* and so you know at the end of *her* day at school the bell rings and the kids all leave and then *she thinks* she can…come here at 4:30 to pick her [child] up, or 4:29, and she’s the…first one here in the morning and the last one to leave…’

Elaborating, she revealed the interconnectedness of self-identity and organizational identity:

‘...it’s a reflection on *my business*…if I don’t *give* her…the *whole* amount of time…she’s gonna be like well it’s so expensive…[or not] sign another contract...bad news travels faster when you’re a business than good news…’

While holding two crying toddlers, she added: ‘I feel it’s a reflection of how giving I am…and it’s *really* hard’. Amy’s combined guilt and frustration, together with her livelihood concerns, demonstrates this work is indeed done for both ‘love and money’ (Folbre & Nelson, 2000).

Like Amy, Alma [Latina-immigrant, family, 40 s] struggled with ‘very emotional’ dilemmas when trying to protect children from perceived parental neglect, while respecting boundaries:

‘It’s hard…you're in between two people, I do the right thing or

stay shy and just turn my face away and…what if something happened, it’s gonna be on

your conscience because you let it go. So there's a lot of emotion going on….’

Providers must therefore creatively balance demands of ‘morality’ and the ‘market’ (Zelizer, 2005). While teachers also bear the burden of conscience, Directors might mediate such situations, shaping the practice of skill in centers distinctly from in family-based care.

## Conclusions

This article challenges the legitimacy of constructed norms about professionalism that promote standardization and devalue emotional labor and capital rather than centering them as vital and credible (Bolton, 2005; Cohen, 2010; Ed. Langford, 2019; Fairchild & Mikuska, 2021; Mikuska & Fairchild, 2020; Morris, 2021; Payne, 2009; Taggart, 2011; Vincent & Braun, 2013). More specifically, drawing on workers’ own emphatic critiques of how credentialing misunderstands skill in ECEC work, this paper challenges the assumption that credentialing requirements are essential for skill-building or enhanced service quality in the sector (Bolton, 2004; Tuominen, 2003; Uttal, 2002).

Instead, it demonstrates that experiential skills are critical across ECEC settings. Workers exercise tacit, embodied and discretionary skills, making autonomous, spontaneous decisions while managing emotions internally and in children and parents. Doing this appropriately and professionally makes the demands of emotional labor particularly challenging. Thus, contrary to the assumption that ECEC work is natural, this paper demonstrates that in both family and center-based settings, work *is* skilled. These skills are *learned*, not natural. They are embedded in the performance of emotional labor and their *practice* is *organizationally shaped* across ECEC settings. Thus, building on existing scholarship, this paper demonstrates that the employment context of care matters and can shape differing requirements for emotional labor (Cohen, 2010; Lopez, 2010; Payne, 2009; Rodriquez, 2014; Vincent & Braun, 2013). Variations across these differently institutionalized forms of care—family and center-based—therefore lie not in the degree of skill, but in its *practice*.

In both centers and family-based care, emotional labor is less supervised and routinized than in other low-wage work (Leidner, 1993). The work performed requires considerable autonomy/discretion and task variety pointing to its skillfulness (Payne, 2009). Teachers and family providers exercise significant control, albeit to different degrees, over how they manage feelings. Autonomy, discretion and task variety manifest through boundary work strategies uniquely shaped by organizational context. These strategies shape context-specific practices of skill in emotional labor.

Findings reflect the tension in Hochschild’s (1983) own definition of emotional labor—as labor-power, with criteria-based qualifications. In discussing what she refers to as paid childcare, Hochschild (1983) notes that this work may not satisfy *all* dimensions of emotional labor. Consistent with this, this paper finds that while workers^15^*do* self-supervise emotions in accordance with client expectations and norms across settings, these feelings are not directly managed. By building upon care-work scholarship that explores autonomy in emotional labor (Lopez, 2006; Paules, 1991; Pierce, 1996; Rodriquez, 2014), this paper demonstrates the latter is part of a feedback loop consisting also of task complexity and experiential skill acquisition, which enables the performance of skill in emotional labor.

By demonstrating how the *practice* (rather than degree) of skill in emotional labor varies across less and more institutionalized ECEC contexts with both children and parents, this paper complicates understandings of gendered skill in emotional labor, illustrating that family-based care is no less skilled than more institutionalized forms of ECEC work. In particular, it situates both center-based and family-based care in the middle of Lopez’s (2006) continuum; while family-based care exhibits more elements of *emotional care* than centers, neither do so fully. Contrary to popular perceptions that devalue family-based care relative to center-based care (Folbre, 2001; Uttal, 2002), family-based providers are no less skilled than teachers. While race has not been the focus of this paper, findings show that there is no evidence that Latina-immigrant women, who predominantly work in family-based care, are ‘naturally’ more caring or less ‘skilled’ than White women (Tuominen, 2003; Uttal, 2002). Instead, irrespective of race and ethnicity, accounts about work and skill in emotional labor are similar across contexts.

More generally, the paper’s findings contribute to scholarship that explores emotional labor in different care settings (Cohen, 2010; Fisher & Tronto, 1990; Lopez, 2006; Nelson et al., 2016; Payne, 2009; Rodriquez, 2014; Stacey, 2011; Vincent & Braun, 2013).

In several other ways, too, this paper contributes to a re-emergent body of scholarship on care labor, emotional labor, professionalization, and credentialing within and beyond the United States (Bolton, 2005; Cohen, 2010; Ed. Langford, 2019; Fairchild & Mikuska, 2021; Mikuska & Fairchild, 2020; Morris, 2021; Osgood, 2010; Payne, 2009; Taggart, 2011; Tuominen, 1998; Uttal, 2002; Vincent & Braun, 2013). It is the author’s hope that the paper’s findings can be used to inform policy debates and advocacy about the limitations that lie in current professionalization standards and norms. This might motivate the realization of more sustainable work requirements for ECEC sector workers, and greater levels of valuation for the complex skills that characterize the emotional labor of ECEC work.

This research also calls for further empirical studies of skill in emotional labor across and within occupations. Particular attention should be given to how race and class shape the gendered construction of skill, interacting with organizational context. It is hoped that this research will inform existing frameworks for conceptualizing emotional labor across care/non-care occupations and organizational contexts. Finally, greater attention must be given to the tension between workers’ conceptions of skill and dominant, gendered skill constructs. By giving visibility to the often invisible skills associated with ECEC work, this research contributes to attempts to challenge the continued devaluation of work in the sector and the assumptions driving gender-based labor market inequalities.

## Data Availability

I collected this data myself.

## Appendix

See Tables 1 and 2.

**Table 1:** Semi-structured interviews: cross-context data

Family-based care providers	Center-based teachers	Non-teachers/providers^a^	Total
16	20	7	43

^a^Non-teachers consist of Directors, one administrative assistant and the primary representative for the Massachusetts State Department for Early Education and Care

**Table 2:** Semi-structured interviews: teachers and providers by race

White	Non-White^a^
27	16

^a^All identified as Latin X, except one who identified as Black

## Footnotes

1 The author recognizes that the global COVID-19 pandemic has changed the role of home-based care in many countries. However, an examination of the implications of that shift is beyond the scope of this paper. The author also notes that in other care contexts like the U.K. the concept of ‘child minding’ is comparable to home-based care. While also beyond the scope of this paper, there is a connection between many forms of ECEC and class (Nixon 2009; Vincent and Braun 2013).

2 A separate paper builds on the same dataset to examine misplaced racialized narratives about care work that suggest Latina women, for instance, are ‘naturally’ more caring than White women. Findings demonstrate that, instead, organizational context shapes how the emotional labor of care is performed, more than race and ethnicity.

3 Vincent and Braun (2013), for example, identify ECEC in the U.K. as a skilled but low autonomy occupation comprised largely of working-class women. While an analysis of class is beyond the scope of this paper, my analysis demonstrates that in the ECEC sector in Massachusetts autonomy is a pre-requisite for skill in emotional labor.

4 While a discussion of gender as it intersects with class is beyond the scope of this paper, Vincent and Braun (2013) show how child care is predominantly performed by young working-class girls.

5 Boundary work emerges in other care and non-care professions (Cain, 2017; Duffy et al., 2015; Osnowitz & Henson, 2016). Cain (2017) finds it gendered in hospices, through emotion management skills.

6 Department of Early Education and Care. Child Care and Development Fund Program. FFY 2022–2024.

7 Massachusetts Department of Early Education and Care 2018: https://www.mass.gov/doc/eecs-guide-to-choosing-child-care-nov-2018.

8 This Massachusetts Department of Early Education and Care developed the state Quality Rating and Improvement System (QRIS) to improve the quality of programs across the state.

9 One follow-up and the state representative interview were on the phone. One interview was not recorded.

10 I found Latina-immigrant women were more likely to link to agencies to minimize linguistic challenges with parents.

11 In a separate paper, I demonstrate that contrary to popular perceptions how organizational context matters more than race or ethnicity in shaping the performance of care and emotional labor.

12 One respondent did not consent to recording; one recording was damaged and two interviews were important but less central to my analysis.

13 It is notable that emotional care was addressed for social workers in the study, but not for ECEC workers, speaking to the differential value given to each (Lopez 2006).

14 Elfer (2012, 2015) demonstrates that when childcare workers feel cared about and supported, they are better equipped to provide effective care.

15 In this paper, I use the term workers to refer to childcare staff in both family and center-based care. I also use the term “providers” for family-based care workers and “teachers” for center-based workers, consistent with the language of the ECEC sector.

## Declarations

### Declarations

This paper complies with all elements of the required declarations guidelines. Consent was obtained from all informants as per the methods section.
